# The Polish Academic Version of the MATRICS Consensus Cognitive Battery (MCCB): Evaluation of Psychometric Properties

**DOI:** 10.1007/s11126-015-9343-9

**Published:** 2015-01-20

**Authors:** Małgorzata Jędrasik-Styła, Agnieszka Ciołkiewicz, Rafał Styła, Magdalena Linke, Dorota Parnowska, Anna Gruszka, Mirella Denisiuk, Marek Jarema, Michael F. Green, Adam Wichniak

**Affiliations:** 1 Third Department of Psychiatry, Institute of Psychiatry and Neurology, Sobieskiego 9 Street, 02-957 Warsaw, Poland; 2 Department of Psychology, Warsaw University, Stawki 5/7 Street, 00-183 Warsaw, Poland; 3 David Geffen School of Medicine, Semel Institute for Neuroscience and Human Behavior, UCLA, 760 Westwood Plaza, Rm 77-361, Los Angeles, CA 90024-1759 USA

**Keywords:** Schizophrenia, Neuropsychology, Neurocognitive function, Cognitive deficits, MATRICS consensus cognitive battery

## Abstract

Work and social functioning in schizophrenia are strongly influenced by cognitive impairment so improving cognition is a priority in the treatment of schizophrenia. Until recently the lack of a widely accepted index of cognitive change for use in schizophrenia was a major obstacle to the development of cognition enhancing treatments. The MATRICS (measurement and treatment research to improve cognition in schizophrenia) consensus cognitive battery (MCCB) was developed as a standard cognitive battery for use in clinical trials of cognition enhancing treatments for schizophrenia and has attracted worldwide interest. To analyze the reliability and validity of a translated and adapted Polish approved academic version of the MCCB. Sixty one patients were assessed at baseline and again after 30 days. The study protocol approximated the MATRICS psychometric and standardization study; the 10 tests that comprise the MCCB were administered to participants. Functioning and psychopathological symptoms were also assessed. Patients and test administrators also assessed the tolerability and practicality of all the cognitive tests. All tests in the battery were found to have high test-retest reliability. All the tests were rated as tolerable and practical by patients and administrators. However practice effects were generally higher in the Polish version of the MCCB than in the original version. Our analysis corroborates previous evidence that the MCCB represents a good tool for assessing cognitive deficits in research studies of schizophrenia also in non-English speaking countries.

## Introduction

Cognitive disorders have been considered to be a central feature of schizophrenia [1, 2] and are now recognized as a distinct feature of the illness [3, 4]. The decrement in cognitive performance affects almost all areas of patients’ cognitive functioning [5], however profiles of cognitive impairment in schizophrenia are not always consistent [6]. The most common cognitive impairments include deficits in attention, aspects of memory, visual-spatial coordination, and executive functioning. In view of the data indicating a strong connection between cognitive impairments and degree of functioning in schizophrenic patients, the presence and seriousness of cognitive symptoms may be considered a crucial factor in determining the progress of the disease [7, 8]. Cognitive deficits account for poor social and professional adaptation and an unfavorable course of the disease in people diagnosed with schizophrenia better than intensification of other symptoms [3, 9].

The assessment of cognitive functioning in clinical trials is clearly important, but there are still remaining concerns about measurement of cognitive impairments. One concerns the use of different tools in different countries which makes comparison of results difficult. In this context the MATRICS (measurement and treatment research to improve cognition in schizophrenia) consensus cognitive battery (MCCB) assumes particular significance. Developed for the assessment of the effects of pharmacological treatments on cognitive performance in schizophrenia [10, 11], it is now being used in countries.

The MCCB has already been translated and adapted into 16 languages (see http://matricsinc.org). Reports on the standardization and normalization of the MCCB battery have confirmed that it is well-suited for use in research and clinical practice in places such as Norway and Spain [12–14]. The good psychometric properties of the MCCB, its practicality and relatively similar results in norming studies make the MCCB useful for cognitive assessment in schizophrenia in different countries. The aim of the current study was to test the psychometric properties of the approved academic Polish translation of the MCCB.

## Materials and Methods

### MCCB Translation and Study Design

All the tests included in the MCCB and a detailed description of their administration and scoring were translated and adapted to Polish conditions, following formal permission from the intellectual property owners. The back translation was sent to the authors of each test in the battery and their comments were carefully analyzed and applied to the translated version. The current study was designed to approximate the MATRICS psychometric and standardization study (MATRICS-PASS; Nuechterlein et al. [11]).

The study was approved by the local ethics committee and was carried out in accordance with the ethical standards laid down in the Declaration of Helsinki. All participants provided written informed consent.

### Participants

Participants included 46 inpatients and 15 outpatients who attended the mental health centre at the same institute. We included participants with an interview-based DSM-IV-TR (Diagnostic and statistical manual of mental disorders–text revised; [15]) diagnosis of schizophrenia who agreed to take part in the research. All the patients enrolled were clinically stable, with no acute psychotic symptoms and no medication changes in the month before or during the study. We excluded patients who had an identified neurological diseases or had a history of head injury, history of alcohol or drug dependence or substance abuse in the past month. Participants did not take sedatives, hypnotic medication, alcohol or psychoactive substances other than prescription medication for 3 days prior to testing.

The patients were assessed at baseline, and again after 30 days. Of the participants 40 were male (66 %) and 21 female. Mean age was 34.43 years (SD = 10.3) with minimum of 18 years and a maximum of 59 years. All participants were white Polish citizens, and their mean educational level was 14 years (SD = 2.7). A one-sample *t* test was used to compare the Polish sample with the original sample from MATRICS-PASS, Polish subjects were significantly younger (p < 0.001, Cohen’s d = 0.93) and better educated (p < 0.001, Cohen’s d = 0.58). All the patients were being treated with antipsychotic medications (olanzapine, aripiprazole, risperidone, amisulpride, clozapine, quetiapine, sertindole, ziprazidone) as monotherapy or in combination with other antipsychotic drugs, antidepressants or mood stabilizers.

### Procedure

The study protocol followed the MATRICS-PASS protocol Nuechterlein et al. [11].

The 10 tests that comprise the MCCB were administered to all participants in the order recommended by the authors of the original MCCB [16].

The order of administration of the MCCB tests was as follows:

1. *Trail making test* Part A (TMT-A)
2. *Brief assessment of cognition in schizophrenia* symbol coding (BACS SC)
3. *Hopkins verbal learning test* revised (HVLT-R)
4. *Wechsler memory scale*––*Third Edition* Spatial span (WMS-III SS)
5. Letter-number span test (LNS)
6. *Neuropsychological assessment battery* Mazes (NAB Mazes)
7. *Brief visuospatial memory test* revised (BVMT-R)
8. *Category fluency test* animal naming (Fluency)
9. *Mayer-Salovey*––*Caruso emotional intelligence test* managing emotions (MSCEIT ME)
10. *Continuous performance test* identical Pairs (CPT-IP)

There is an alternative version of 3 of the 10 tests: HVLT-R, BVMT-R and NAB Mazes that were used in the MATRICS-PASS study. As recommended in the MCCB manual, Version 4 of HVLT-R and Version 5 of BVMT-R were included into the study for repeated testing. NAB Mazes has only one alternative version, which was used. The order of use of the alternative forms was counterbalanced across and within subjects.

Participants and test assistants assessed the tolerability and practicality respectively of all the tests on a seven-point Likert scale. As well as making a global assessment of practicality, testers rated separately the ease of setting up the test, its administration, and scoring.

As we found significant differences in the administrators’ ratings, particularly for ratings of administration and scoring, test assistants rated the battery after administering the battery to each of the patient. Hence, the scores reflected the administrators’ impression of the testing experience, which is broader than the ratings of the original version that only reflected the testers’ cumulative judgment of a test’s practicality.

Participants provided a subjective rating of the tolerability of the tests. Immediately after completion of the battery participants were asked to rate (1) how unpleasant or pleasant and (2) how difficult or easy they found each test on a seven-point Likert scale. It was anchored by happy and unhappy faces (coded 7 and 1 respectively) corresponding to the level of pleasantness.

The positive and negative syndrome scale (PANSS) for assessment of psychiatric symptoms [17], global assessment of functioning (GAF) (described in the DSM-IV-TR) and strauss-carpenter level of function scale (SCLOF) [18, 19] for the assessment of community and work functioning were also completed for all participants.

## Results

### Test–Retest Reliability

Test–retest reliability data are summarized in Table 1. Test retest reliability was highest for BACS SC, and the lowest for the WMS-III SS. All the tests were in the acceptable range. The composite score is a mean of the standardized scores for each of the seven cognitive domains. The test–retest reliability for the composite score was very high, r (59) = 0.93, p < 0.001.

**Table 1:** Test-retest reliability

Tests	Test scores used	Pearson’s coefficient (r)
Speed of processing		
Category fluency test, animal naming	Total number of animals named in 60 s	0.71
Trail making test, Part A (TMT-A)	Time to completion	0.80
Brief assessment of cognition in schizophrenia, symbol coding subtest (BACS SC)	Total number correct	0.91
Attention/vigilance		
Continuous performance test––identical pairs version (CPT-IP)	Mean d’ value across 2-, 3-, and 4-digit conditions	0.83
Working memory		
Letter-number span test (LNS)	Number of correct trials	0.73
Wechsler memory scale, spatial span subtest (WMS-III SS)	Sum of raw scores on forward and backward conditions	0.69
Verbal learning		
Hopkins verbal learning test––revised, immediate recall (HVLT-R)	Total number of words recalled correctly over three learning trials	0.80
Visual learning		
Brief visuospatial memory test-revised (BVMT-R)	Total recall score over 3 learning trials	0.78
Reasoning and problem solving		
Neuropsychological assessment battery, mazes subtest (NAB Mazes)	Total raw score	0.74
Social cognition		
Mayer-Salovey-Caruso emotional intelligence test, managing emotions branch (MSCEIT ME)	Branch score using general consensus scoring	0.82

### Utility as a Repeated Measure

The utility evaluation comprised (1) the mean difference in test’s scores across two occasions, (2) statistical significance of these differences, (3) effect size of the change (magnitude of change was assessed with Cohen’s d; this calculates how much the follow-up result (T_2_) deviates from the baseline (T_1_), it was calculated using following formula: effect size = (T_1_−T_2_)/SD_1_ and 4) the number of subjects achieving the maximum and minimum score. The results are presented in Table 2.

**Table 2:** Practice effects of the MATRICS battery––polish version: performance levels at baseline (T_1_) and follow-up (T_2_)

Tests	T_1_ mean (SD)	T_2_ mean (SD)	T_2_−T_1_ mean difference (SD)	*t* test (p value)	d-Cohen effect size	Floor/ceiling effect (n out of 61 in T_1_ and T_2_)
Speed of processing						
Fluency	22.1 (6.5)	23.9 (6.3)	1.8 (4.9)*	2.85 (p < 0.01)	0.27	0/0
TMT-A	40.6 (16.8)	34.8 (14.1)	−5.8 (10.1)*	−4.52 (p < 0.001)	0.35	0/0
BACS SC	45.0 (10.9)	47.9 (11.0)	2.9 (4.5)*	4.91 (p < 0.001)	0.26	0/0
Attention/vigilance						
CPT-IP	2.1 (0.7)	2.3 (0.7)	0.2 (0.4)*	3.85 (p < 0.001)	0.29	0/0
Working memory						
LNS	13.6 (3.2)	14.4 (3.1)	0.8 (2.3)	2.66 (p = 0.01)	0.24	0/1
WMS-III SS	16.0 (3.4)	15.6 (3.6)	−0.4 (2.7)	−0.89 (p = 0.38)	0.09	0/0
Verbal learning						
HVLT-R	23.3 (6.1)	24.9 (5.9)	1.6 (3.8)*	3.23 (p < 0.01)	0.26	0/0
Visual learning						
BVMT-R	24.5 (7.5)	26.7 (7.5)	2.2 (4.9)*	3.51 (p < 0.001)	0.29	0/1
Reasoning and problem solving						
NAB Mazes	15.1 (6.2)	17.5 (6.7)	2.4 (4.7)*	3.95 (p < 0.001)	0.38	0/5
Social cognition						
MSCEIT ME	87.6 (10.6)	89.5 (10.2)	1.9 (6.2)*	2.34 (p = .02)	0.17	0/0

* Mean T_2_−T_1_ difference is statistically significantly different from the original American sample [16]

The changes in tests’ scores across two occasions reached statistical significance for 8 out of 10 tests, the exceptions were WMS-III SS and MSCEIT-ME. Most of the tests showed a small practice effect. The largest practice effect was obtained for the NAB Mazes test (for which the improvement was 0.38), whereas the smallest effects were observed for the working memory measures. There were no detectable ceiling effects.

### Relationship with Functional Outcome

We also examined the ecological validity of the Polish version of the MCCB. The correlations between test results and functional status measures were computed to assess the relationship between cognitive performance as measured by the Polish MCCB and functioning. Correlations between the composite and 10 MATRICS test scores, and GAF and SCLOF scores are presented in Table 3.

**Table 3:** Relationship between cognitive performance and functioning

Correlations						
	Level of function scale (SCLOF)					Global assessment of functioning (GAF)
Domain and tests	Global	Work subscale	Social contact subscale	Functioning subscale	Symptomatology subscale	
Composite score	0.47**	0.35**	0.40**	0.47**	0.33*	0.49**
Speed of processing						
Fluency	0.23	0.18	0.16	0.25	0.14	0.22
TMT-A	−0.49**	−0.33*	−0.47**	−0.50**	−0.31*	−0.44**
BACS SC	0.41**	0.34**	0.31*	0.37**	0.33*	0.42**
Attention/vigilance						
CPT-IP	0.33**	0.21	0.32*	0.38**	0.17	0.36**
Working memory						
LNS	0.44**	0.27*	0.41**	0.38**	0.38**	0.44**
WMS-III SS	0.38**	0.38**	0.27*	0.36**	0.24	0.39**
Verbal learning						
HVLT-R	0.28*	0.17	0.25*	0.24	0.27*	0.23
Visual learning						
BVMT-R	0.20	0.09	0.33*	0.16	0.07	0.24
Reasoning and problem solving						
NAB Mazes	0.34**	0.32**	0.26*	0.36**	0.15	0.30*
Social cognition						
MSCEIT ME	0.32*	0.25	0.13	0.37**	0.30**	0.38**

* p < 0.05, ** p < 0.01

The global cognitive performance (MATRICS composite score) correlate largest with global score of both functioning scales (GAF, SCLOF global score) and SCLOF functioning subscale (which covers fullness of life and overall level of function). The smallest correlations with cognitive performance were found in two the SCLOF domains: work and symptomatology, where cognitive performance explained roughly 10 % of the variance. There were higher correlations between functioning and tests of working memory and for two of the three tests of speed of processing; the largest correlation was 0.5 for TMT-A and the smallest correlation was with fluency.

### Practicality and Tolerability

The practicality ratings (of administrators) and tolerability ratings (of participants) are presented in Table 4 together with the mean time needed to administer each test. Most tests were considered to be both practical and tolerable. The most complicated to set up of the 10 tests was WMS-III SS. This was also regarded as the most difficult to administer. All the tests were rated as very easy to score, WMS-III SS and MSCEIT being the most difficult, although still relatively easy. All the tests required between 1.78 and 14.68 min to administer, with CPT-IP and MSCEIT-ME taking longest. The mean total time needed to administer the Polish version of MCCB was 85.12 min including pauses, corresponding to 70.48 min of actual test administration time.

**Table 4:** Practicability and tolerability of the 10 tests of the MATRICS battery

Domain, and tests	Practicality				Tolerability		
	Global	Setup	Administration	Scoring	Participant ratings: difficult–easy	Participant ratings: pleasant–unpleasant	Administration time (minutes)
	Mean (SD)	Mean	Mean	Mean	Mean	Mean	Mean
Speed of processing							
Fluency	6.51 (0.63)	6.95	6.00	6.58	5.59	5.74	1.78
TMT-A	6.96 (0.14)	6.97	6.93	6.98	6.23	6.39	2.14
BACS SC	6.89 (0.23)	6.92	6.92	6.85	5.31	5.70	2.76
Attention/vigilance							
CPT-IP	6.56 (0.74)	6.43	6.55	6.68	3.95	4.66	13.78
Working memory							
LNS	6.55 (0.69)	6.68	6.33	6.63	3.77	4.70	6.86
WMS-III SS	5.77 (0.69)	6.33	5.00	5.98	4.28	5.11	6.96
Verbal learning							
HVLT-R	6.61 (0.58)	6.78	6.38	6.65	4.02	4.70	4.55
Visual learning							
BVMT-R	6.37 (.69)	6.62	6.35	6.13	3.98	4.89	5.57
Reasoning and problem solving							
NAB Mazes	6.42 (0.67)	6.62	6.12	6.53	4.44	5.33	11.4
Social cognition							
MSCEIT ME	6.00 (0.86)	6.53	6.38	6.08	4.52	4.89	14.68

## Discussion

### Test–Retest Reliability

Immediate test–retest reliability (short-term stability) was judged by the MATRICS Neurocognition Committee to be the feature of the test most relevant to use in clinical trials; it is important that the test reflects actual changes in cognitive functioning rather than measurement error, practice effects or other factors related to exposure to the test situation, test materials or administrator. Most cognitive deficits in schizophrenia are stable over time in the absence of specifically designed intervention [4], so a four-week re-test interval in clinically stable patients should provide good indication of the stability of the tests. Data from the current study found high test–retest reliability for all the MCCB tests. The MATRICS Committee considered an r value of 0.70 to represent acceptable test–retest reliability for clinical trials. Most of the tests achieved at least that level and many showed even higher stability; test–retest reliability varied from 0.69 for WMS-III SS to 0.91 for BACS SC. Comparable results (r’s between 0.69 and 0.85) were obtained in an PASS study [11]; in that study the BACS SC also had the highest test–retest reliability.

### Utility as Repeated Measure

Tests are considered useful for clinical trials if they are subject to relatively small practice effects. This characteristic is crucial when a test is administered several times. If there are detectable practice effects, the maximum score should very rarely be obtained [20]. Practice effects were generally higher in the Polish version of MCCB than the original version. Differences between the Polish and American samples may partly explain the larger practice effects found in the current study. The samples differed significantly in age and education. A one-sample *t* test showed that the Polish subjects were younger and better educated. Other factor which may have contributed to the difference is an effect of hospitalization on cognitive improvement. Although the entire Polish sample met the criterion of no changes in pharmacological treatment used in the American study, in the month before the assessment 46 of the participants took part in an intensive psychotherapeutic program as a part of a rehabilitation program. Although this did not involve any training specifically aimed at cognitive functioning, participation in everyday classes involved attention and memory processes and may have stimulated cognitive functions. Cognitive improvements might be expected after hospital discharge as participants have a more active social and occupational life; some of our patients were discharged during the study.

Even with the observed practice effects there was still substantial room for additional improvement. Maximum scores were very rarely obtained on any of the MCCB tests, and test showed a floor effect. The Polish MCCB may therefore be seen as a feasible measurement of cognitive change with treatment over time. Because practice effects are usually largest between the first and second administrations, multiple baseline assessments session might be an effective way to reduce practice effects.

### Relationship with Functional Measure

The overall magnitude of the correlations between the Polish MCCB test scores and functional status scores provides evidence for an association between performance on this battery tests and functioning in schizophrenia. However the outcome pattern we found is different from that obtained in other studies [21, 22], where correlations between MCCB test score and work functioning were larger and correlations between MCCB test score and social outcomes were smaller. We found that correlations between MCCB test score and functioning were smallest for the work and symptomatology SCLOF domains.

The relatively weak association between cognitive functioning and symptoms which we observed is consistent with other reports [4]. The weak relationship between work and cognitive functioning in our study might be explained by cultural and social factors specific to Polish communities, where high unemployment reduces disabled peoples’ access to employment and other opportunities for occupational activities are low [23].

In this context the results of Gold et al. [22] which suggested that cognitive performance was a good predictor of job tenure but not job attainment should be noted. These authors found no differences in cognitive performance between subjects who obtained paid employment and those who remained unemployed during the follow-up period. In contrast, they found that multiple cognitive measures (including measures of IQ, attention, working memory and problem solving) were significantly correlated with the duration of employment. Although there is substantial evidence for associations between cognition and functional outcome in schizophrenia, functioning is also affected by other factors usually not considered in clinical trials e.g. psychosocial rehabilitation and educational and vocational opportunities (see: Green et al. [10, 24]).

### Practicality, Tolerability, Administration Time

Ease of use is an important characteristic of tests designed to measure changes in cognition. The overall practicality score fell above the scale midpoint, scores for individual tests ranged from 5.77 for the WMS-III SS to 6.96 for the TMT-A. MCCB may therefore be described as relatively simple to set up, administer and score. The rating of the WMS-III SS is because it demands significant attention from the administrator as well as requiring additional equipment.

Tolerability is considered a crucial characteristic of tests that may be administered to a subject multiple times. Patients rated the tests of the Polish MCCB relatively highly on a scale of pleasantness, with mean scores ranging from 4.70 for LNS and HVLT-R to 6.39 for the TMT-A. LNS and HVLT-R were designed to measure the efficiency of memory processes and are demanding and easily associated with the school exam situation. This aspect may result in the patient being confronted with his or her difficulties in a very explicit manner. The TMT-A on the other hand measures psychomotor performance and is quick and easy to do.

The MATRICS Neurocognition Committee indicated that battery of tests intended for repeated administration in clinical trials should provide a reliable, valid assessment of all relevant cognitive domains within 60–90 min [16]. In this study of the 10 Polish MCCB tests the total administration time for the battery was 70.48 min of actual test administration time, in the middle of the range of durations viewed as acceptable by experts.

In summary, the MCCB is characterized by high relative reliability. All the tests of the Polish MCCB were rated positively by people suffering from schizophrenia and test administrators, being considered both practical and tolerable. The time required to administer the whole test battery is reasonable. This study, conducted on a Polish sample of patients with schizophrenia, has proved that the Polish version of the MCCB is a valuable instrument for assessment of cognitive functions for clinical and research purposes in Polish communities.
